# Meta-analysis of extracorporeal membrane oxygenation in combination with intra-aortic balloon pump vs. extracorporeal membrane oxygenation only in patients with cardiogenic shock due to acute myocardial infarction

**DOI:** 10.3389/fcvm.2022.1104357

**Published:** 2023-01-18

**Authors:** Max M. Meertens, Tobias Tichelbäcker, Sascha Macherey-Meyer, Sebastian Heyne, Simon Braumann, Stephan F. Nießen, Stephan Baldus, Christoph Adler, Samuel Lee

**Affiliations:** Faculty of Medicine, Clinic III for Internal Medicine, University Hospital Cologne, University of Cologne, Cologne, Germany

**Keywords:** VA-ECMO, IABP, cardiogenic shock, acute coronary syndrome (ACS), meta-analysis

## Abstract

**Background:**

Incidence and mortality of cardiogenic shock (CS) in patients with acute myocardial infarction (AMI) remain high despite substantial therapy improvements in acute percutaneous coronary intervention over the last decades. Unloading the left ventricle in patients with Veno-arterial extracorporeal membrane oxygenation (VA-ECMO) can be performed by using an intra-aortic balloon pumps’ (IABP) afterload reduction, which might be especially beneficial in AMI patients with CS.

**Objective:**

The objective of this meta-analysis was to assess the effect of VA-ECMO + IABP vs. VA-ECMO treatment on the mortality of patients with CS due to AMI.

**Methods:**

A systematic literature search was performed using EMBASE, COCHRANE, and MEDLINE databases. Studies comparing the effect of VA-ECMO + IABP vs. VA-ECMO on mortality of patients with AMI were included. Meta-analyses were performed to analyze the effect of the chosen treatment on 30-day/in-hospital mortality.

**Results:**

Twelve studies were identified by the literature search, including a total of 5,063 patients, 81.5% were male and the mean age was 65.9 years. One thousand one hundred and thirty-six patients received treatment with VA-ECMO in combination with IABP and 2,964 patients received VA-ECMO treatment only. The performed meta-analysis showed decreased mortality at 30-days/in-hospital after VA-ECMO + IABP compared to VA-ECMO only for patients with cardiogenic shock after AMI (OR 0.36, 95% CI 0.30–0.44, *P*≤0.001). Combination of VA-ECMO + IABP was associated with higher rates of weaning success (OR 0.29, 95% CI 0.16–0.53, *P* < 0.001) without an increase of vascular access complications (OR 0.85, 95% CI 0.35–2.08, *P* = 0.72).

**Conclusion:**

In this meta-analysis, combination therapy of VA-ECMO + IABP was superior to VA-ECMO only therapy in patients with CS due to AMI. In the absence of randomized data, these results are hypothesis generating only.

## Introduction

Cardiogenic shock (CS) describes a clinical condition of inadequate tissue or end-organ perfusion due to acute heart failure. The predominant cause of CS is acute myocardial infarction (AMI). CS occurs in 5–10% of patients with AMI and is associated with very poor prognosis (30-day mortality of approximately 40%) (1). In the United States, 40,000–50,000 patients are suffering from CS each year and incidence of cardiogenic shock in Europe is rising (2, 3). Despite several improvements in treatment of AMI patients in the last two decades including new stent technologies, reperfusion strategies, or pharmacological options, mortality rates in CS are still unacceptably high (4).

Mechanical circulatory support (MCS) systems, such as veno-arterial extracorporeal membrane oxygenation (VA-ECMO), were implemented in clinical practice at specialized cardiac arrest centers to improve prognosis of CS patients (5). The European Society of Cardiology guidelines give the use of MCS in CS a IIa C recommendation (6). Therefore, VA-ECMO therapy is widely used in patients with CS (2, 7). However, this approach lacks robust clinical evidence based on randomized data and entails disadvantageous hemodynamics in CS. Retrograde flow in the aorta through the aortic cannula increases left ventricular (LV) afterload which leads to increased LV enddiastolic pressure, left atrial pressure, and pulmonary capillary wedge pressure. This results in a worsening of blood oxygen saturation, an increase in myocardial oxygen demand, and a deterioration of coronary circulation which consequently leads to worsening of myocardial infarction (8). Therefore, the AMI population differs substantially from other VA-ECMO treated patients and so called “venting” strategies may play an essential role in treatment of AMI patients with VA-ECMO.

Due to these potentially harmful effects of VA-ECMO therapy especially in patients with CS due to AMI, mainly two “venting” strategies are being used to unload the left ventricle: reducing LV preload with ECMO + a transvalvular microaxial flow pump (Impella, Abiomed, Danvers, MA, USA) and reducing LV afterload with ECMO + an intra-aortic balloon pump (IABP) (9). Combination of VA-ECMO + transvalvular microaxial flow pump is currently investigated in a randomized controlled trial (RCT) in CS patients (10). As there is no RCT investigating VA-ECMO + IABP vs. VA-ECMO only, we conducted this meta-analysis to explore whether combination of VA-ECMO + IABP may benefit patients in CS due to AMI compared to VA-ECMO only therapy.

## Materials and methods

### Literature search and study selection

The systematic review was performed according to a pre-specified protocol and an explicit, reproducible plan for literature research and synthesis as demanded by the Preferred Reporting Items of Systematic Reviews and Meta-Analyses (PRISMA) guidelines (11). The Medline, Cochrane and Embase databases were searched by two authors (MM and TT) in September 2021. Results were updated again on December 11, 2022. The following mesh terms and medical subheadings were used: st elevation myocardial infarction, acute myocardial infarction, myocardial infarction, myocardial ischemia, myocardial infarction [MeSH Term], STEMI, IABP, intra-aortic balloon pumping, intraaortic balloon pumping, intraaortic balloon pumping, counterpulsation device, extracorporeal life support, extracorporeal membrane oxygenation, extra corporeal membrane oxygenation, ecmo [MeSH Term], ECMO, cardiogenic shock, and cardiogenic shock [MeSH Term].

The study selection was conducted by two reviewers (MM and TT). In case of a disagreement, this was solved by consensus with the senior author (SL). Retrieved records retrieved were screened using the title and abstract by both researchers. Eligibility assessment of the full text was considered if the title or abstract seemed eligible. The applied in- and exclusion criteria are described below. The database search was supplemented by scanning the references of the included studies. Irrespective of the study design all studies were included in the systematic review and meta-analysis, if they met the following inclusion criteria: (1) A full text had to be accessible, (2) the studied participants had to be human patients, (3) studies had to involve patients treated for cardiogenic shock after myocardial infarction, even if it was just a subgroup, and (4) studies had to include patients treated by VA-ECMO and patients treated by VA-ECMO + IABP. We excluded studies which did not report absolute values for in-hospital/30-day mortality for both groups.

### Data collection

Eligible data was retrieved by a researcher and verified by a second one for every study (MM and TT). We extracted the following data from each study: first author, country of the institution of the first author, year of publication, study design, size of study population, mean age, percentage of male patients, prevalence of hypertension, diabetes mellitus, peripheral arterial disease, prior stroke, ischemic heart disease, number of implanted stents, location of culprit lesion, reason for IABP usage, number of patients treated with VA-ECMO, and number of patients treated with VA-ECMO + IABP. For both groups we collected the in-hospital/30-day mortality and the incidence of ischemic and bleeding complications. The methodological quality of all included studies was assessed using the checklist recommended by the 9-point Newcastle–Ottawa scale (NOS) (12).

### Statistical analysis

Meta-analyses were performed to compare in-hospital/30-day mortality and incidence of ischemic and bleeding complications of patients treated with VA-ECMO and of patients treated with VA-ECMO + IABP. Fixed effects meta-analyses were performed using the Mantel–Haenszel method for dichotomous data to estimate the pooled odds ratio (OR). Statistical heterogeneity was assessed using *I*^2^ statistics. An *I*^2^ value of more than 75% was considered to indicate a significant heterogeneity. A possible publication bias was assessed and represented by a funnel plot. *P*-values < 0.05 were considered statistically significant. The meta-analysis was performed using in RevMan (version 7.3).

## Results

**FIGURE 1:**
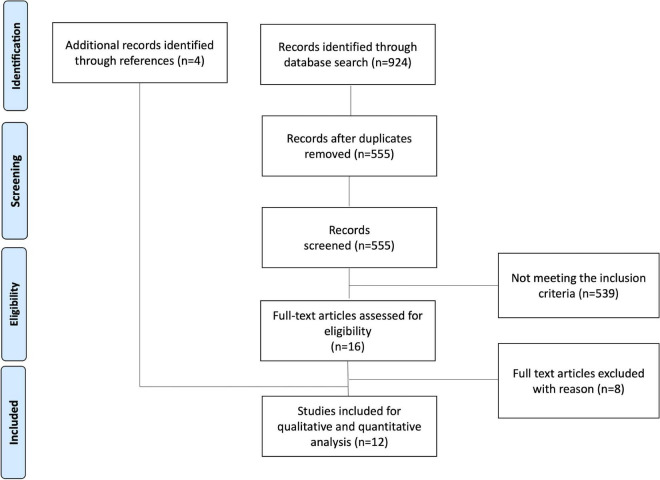
Search flow chart according to PRISMA guidelines.

The search strategy led to 924 results in December 2022 after updating the initial search from September 2021. Removal of duplicates resulted in 555 studies potentially eligible for analysis. Another 535 were removed, because they did not meet the inclusion criteria. Five studies were removed after full text analysis due to lack of explicit mortality data for subgroups or because all patients were treated with VA-ECMO + IABP (13–17). Two more were removed as an ECMO only strategy was compared to ECMO + IABP/Impella or + IABP/pVAD (18, 19). One further registry from Japan was excluded as it included less patients than the Japanese registry study we included (20). Four studies were identified by additional records. Finally, 12 studies were included for qualitative and quantitative analysis (Figure 1; 21–32). None of these were randomized controlled trials but 11 were case series and one was a national register (Table 1). Seven studies were conducted in Asia (21–25, 29, 30), three in Europe (26, 28, 32), and one in the US and Australia (27, 31). In total 5,063 patients with cardiogenic shock after myocardial infarction were included, 1,136 patients were treated with VA-ECMO and 3,927 received VA-ECMO and IABP. The methodological quality of all included studies according to the 9-point Newcastle-Ottawa scale is presented in Table 1. All studies showed an acceptable score.

**TABLE 1:** Assessment of the Newcastle Ottawa scale.

Study	Representativeness of the exposed cohort	Ascertainment of exposure	Ascertainment of exposure	Outcome of interest not present at the start of study	Cohorts comparable on the basis of design or analysis	Assessment of outcome	Adequacy of duration of follow-up	Adequacy of completeness of follow-up
Aoyama	*	*	*	*		*	*	*
Chen	*	*	*	*		*	*	*
Chung		*	*	*		*	*	*
Kagawa	*	*	*	*		*	*	*
Mueller	*	*	*	*		*	*	*
Overtchouk	*	*	*	*		*	*	*
Park	*	*	*	*		*	*	*
Sakamoto	*	*	*	*		*	*	*
van den Brink	*	*	*	*		*	*	*
Xu	*	*	*	*		*		
Kuroki	*	*	*	*	*	*	*	*
Nishi	*	*	*	*		*	*	*

*The study fulfilled the criteria.

### Patient characteristics

The overall mean patient age was 65.9 years and ranged from 53 to 72 years. 81.5% were male, 24.7% of the patients had a history of arterial hypertension and 19.9% had a history of diabetes mellitus type 2. The patient characteristics are summarized in Table 2.

**TABLE 2:** Patient and study characteristics.

First author	Year	Country	Study design	Patiens (n)	ECMO (n)	ECMO + IABP (n)	Mean age	Male	AHT	DM	CAD	Pre. stroke	Pre. MI
Aoyama et al. (21)	2014	Japan	Retrospective cohort	38	3	35	60	88	NC	NC	NC	NC	NC
Chung et al. (22)	2011	Korea	Retrospective cohort	20	6	14	61	70	45	35	NC	NC	NC
Kagawa et al. (25)	2012	Japan	Retrospective cohort	76	15	61	63	70	54	27	NC	NC	19
Muller et al. (26)	2016	France	Retrospective cohort	138	42	96	55	80	NC	NC	NC	NC	NC
Negi et al. (27)	2016	US	Retrospective cohort	15	6	9	57	60	NC	NC	NC	NC	NC
Overtchouk et al. (28)	2016	France	Retrospective cohort	106	43	63	53	84	39	21	23	NC	22
Park et al. (29)	2014	Korea	Retrospective cohort	96	55	41	65	76	23	31	19	NC	12
Sakamoto et al. (30)	2012	Japan	Retrospective cohort	98	4	94	72	66	45	35	NC	9	6
Van den Brink et al. (32)	2021	Netherland	Retrospective cohort	18	11	7	60	78	32	NC	17	NC	NC
Xu et al. (31)	2016	Australia	Retrospective cohort	16	11	5	62	62	NC	38	NC	NC	NC
Kuroki et al. (23)	2021	Japan	Retrospective cohort	627	89	538	65	84	NC	NC	NC	NC	NC
Nishi et al. (24)	2022	Japan	National registry	3,815	851	2,964	67	82	21	19	NC	NC	NC

Vales are presented in percentages unless indicated otherwise. AHT, arterial hypertonus; CAD, coronary artery disease; DM, diabetes mellitus type 2; pre., previous.

### Reasons for IABP treatment

Five studies reported the indications for additional IABP treatment (21, 22, 28, 30, 33). Two studies reported the decision for additional IABP treatment was made by the physician in charge (28, 33), two studies reported that an IABP was routinely implanted in absence of any contra indication (21, 30) and one study reported that an additional IABP was implanted in case the arterial pulse wave disappeared after using VA-ECMO support (22).

### Mortality VA-ECMO + IAPB vs. VA-ECMO at 30-day/in-hospital

**FIGURE 2:**
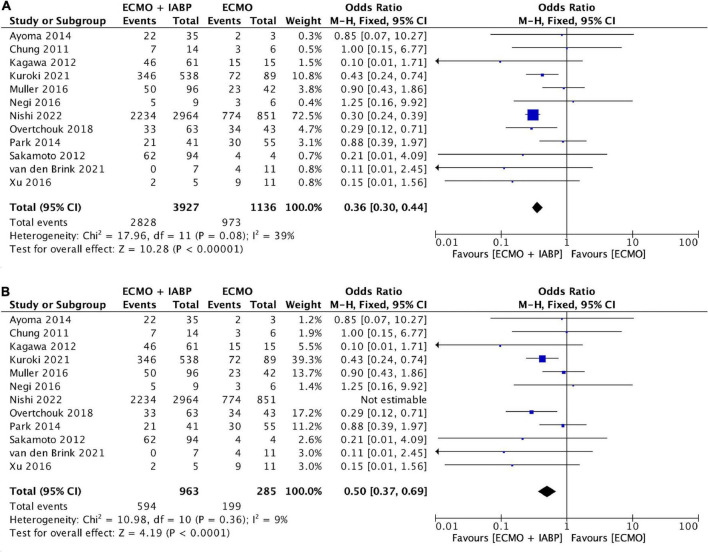
Forest plot on 30-day survival ECMO + IABP vs. ECMO. **(A)** All included studies. **(B)** Subgroup analysis excluding Nishi et al. from 2022. Individual studies and pooled analysis showing a significant decreased 30-day mortality for patients in cardiogenic shock due to acute myocardial infarction treated with ECMO + IABP compared to patients with ECMO only (*p* = 0.003). CI, confidence interval; OR, odds ratio.

**FIGURE 3:**
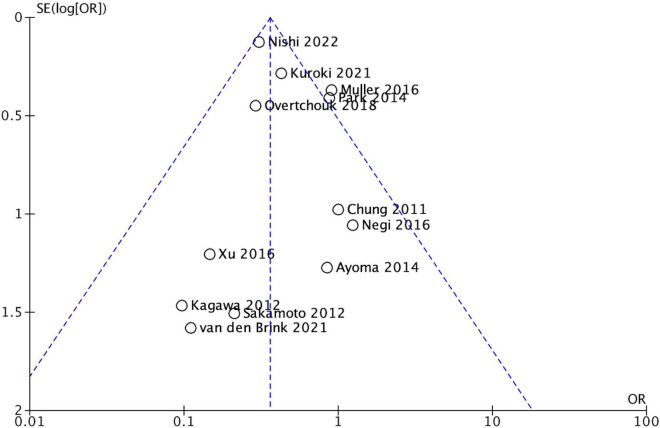
Funnel plot.

Four studies reported the 30-day/in-hospital mortality (23–25, 28) and eight studies the in-hospital mortality (21, 22, 26, 30–33). Overall mortality was 75.1% (*n* = 3802) and 85.6% (*n* = 973) in the VA-ECMO and 72% (*n* = 2829) in the VA-ECMO + IABP group. The in-hospital/30-day mortality was reported in all studies, the conducted meta-analysis showed that patients treated with VA-ECMO + IABP had a significantly lower odds ratio for mortality (OR 0.36, 95% CI 0.30–0.44, *P* ≤ 0.001; Figure 2A), with low heterogeneity (*I*^2^ = 11%). A performed funnel plot showed that the study by Muller et al. might be at risk for reporting bias (Figure 3). A subgroup analysis was conducted in which the Japanese registry by Kuroki et al. was excluded to prohibit including patients multiple times. The overall 30-day/in-hospital mortality within the subgroup analysis including 1,248 patients (ECMO *n* = 285; ECMO + IABP 963) was 63.6% (*n* = 794), in the VA-ECMO 69.8% (*n* = 199) and in the VA-ECMO + IABP group 61.8% (*n* = 595) (Figure 2B).

### Weaning from VA-ECMO + IAPB vs. VA-ECMO

**FIGURE 4:**
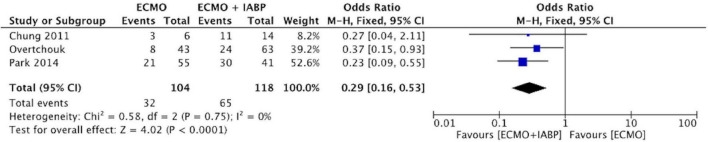
Forest plot on weaning success of ECMO + IABP vs. ECMO. Individual studies and pooled analysis showing a significant increased successful ECMO weaning for patients in cardiogenic shock due to acute myocardial infarction treated with ECMO + IABP compared to patients with ECMO only (*p* < 0.001). CI, confidence interval; OR, odds ratio.

Three studies reported on their successful VA-ECMO weaning rate, including 104 VA-ECMO and 118 VA-ECMO + IABP patients with a success rate of 30.8 and 55.1% (22, 28, 33). Successful weaning was not specifically defined in any of the studies. The conducted meta-analysis of successful VA-ECMO weaning rate showed that patients treated with IABP had a significantly higher odds ratio for successful weaning (OR 0.29, 95% CI 0.16–0.53, *P* < 0.001; Figure 4), with low heterogeneity (*I*^2^ = 0%).

### Vascular complications VA-ECMO + IAPB vs. VA-ECMO

**FIGURE 5:**
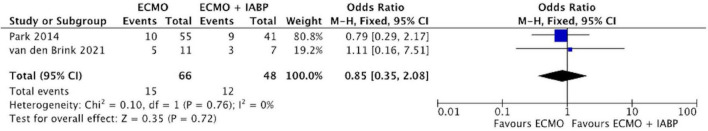
Forest plot on vascular complications of ECMO + IABP vs. ECMO. Individual studies and pooled analysis showing no significant differences in the incidence of vascular complications for patients in cardiogenic shock due to acute myocardial infarction treated with ECMO + IABP compared to patients with ECMO only (*p* < 0.001). CI, confidence interval; OR, odds ratio.

Two studies reported on their vascular complication rate, including 66 VA-ECMO and 48 VA-ECMO + IABP patients with a vascular complication rate of 22.7 and 25% (32, 33). The conducted meta-analysis of the vascular complication rate showed that ECMO only did not significantly lower the risk for vascular access complications (OR 0.85, 95% CI 0.35–2.08, *P* = 0.72; Figure 5), with low heterogeneity (*I*^2^ = 0%).

## Discussion

In patients with CS due to AMI combination of VA-ECMO + IABP was associated with a lower 30-day/in-hospital mortality rate compared to VA-ECMO alone while access site complications were comparable in both groups.

To date, treatment of patients with CS caused by AMI with VA-ECMO is restricted to specialized cardiac arrest centers, and patient selection or indication is not standardized. However, it is uncertain if patients in CS benefit from VA-ECMO compared to optimal medical treatment. We await randomized data of the ECLS shock trials providing data to answer this question. The recently presented ECMO-CS trial showed no benefit of early VA-ECMO implantation without a venting strategy in 117 randomized patients in CS (34), with a crossover rate of 39%. A meta-analysis of retrospective studies showed that VA-ECMO improved 30-day mortality by 13% for patients in CS (35). The randomized ANCHOR trial enrolled its first patient in October 2021 comparing optimal medical treatment with VA-ECMO + IABP in CS due to AMI patients (36).

In this study we conducted the first meta-analysis in the subgroup of CS AMI patients comparing VA-ECMO + IABP vs. VA-ECMO alone. In 2018, a similar meta-analysis was performed in general CS patients and investigated a subgroup of AMI patients (37) in contrast to our trial, this study included all forms of cardiogenic shock. Vallabhajosyula et al. found no impact on survival of a concomitant use of IABP and VA-ECMO in the total cohort. Only the subgroup of AMI patients, not further defined, displayed lower mortality rates in the VA-ECMO + IABP group compared to VA-ECMO alone (OR 0.56). A similar meta-analysis focusing on left ventricular unloading during extracorporeal membrane oxygenation published by Russo et al. in 2019 also showed lower mortality in patients receiving ECMO + IABP in CS compared to ECMO alone. However, they performed no analysis on patients in CS due to AMI (38). In our meta-analysis, conducted with AMI patients only, we were able to add four more trials with a total of 248 patients. Regarding mortality rates, these results were confirmed by our study as VA-ECMO + IABP was associated with lower 30-day/in-hospital mortality rates and comparable effective size (OR 0.36).

Our meta-analysis also showed that VA-ECMO weaning success was superior in patients receiving an additional IABP. The benefit might be a general effect of improved myocardial recovery; additionally, IABP could facilitate a smoother transition between the lowest ECMO to zero flow. Especially in myocardial infarction it might be relevant to make use of a venting strategy as early as possible to counteract an otherwise increased oxygen expenditure of the failing cardiomyocytes (39).

For both IABP and VA-ECMO, patients’ vascular complications are common and might contribute to the overall prognosis. Nearly a third of VA-ECMO and 15% of IABP patients suffer a vascular complication (40, 41). As vascular complications significantly impact mortality in VA-ECMO patients, any additional complications of arterial canulation for IABP would have to be taken very seriously. In patients receiving an VA-ECMO + transvalvular microaxial flow pump, an increase in severe bleeding complications and access site related ischemia was reported (42). Within our meta-analysis, we found no differences regarding access site complications, but only two studies reported data which could be included. This suggests a form of reporting bias. Thus, it is not possible to definitively answer this question based on the included sample size. However, in the IABP Shock II trial there were no differences between the IABP and the optimal medical treatment groups regarding bleeding complications and peripheral ischemia requiring intervention (43). An advantage of IABP compared to a transvalvular microaxial flow pump as a venting device might thus be a lower vascular complication profile due to its smaller access size.

It remains unclear which “venting” strategy might be the best in this special setting. VA-ECMO + transvalvular microaxial flow pump (so called “ECMELLA”) was investigated in a retrospective multicenter study with 686 CS patients and was associated with a lower mortality rate after 30 days compared to VA-ECMO therapy alone (44). In the vast majority of patients, CS was caused by AMI and only about one third was non-ischemic. However, the favorable effect of VA-ECMO + transvalvular microaxial flow pump was consistent in both groups. One further propensity-matched retrospective cohort study reported reduced in-hospital mortality and increased VA-ECMO weaning success in patients with VA-ECMO + transvalvular microaxial flow pump compared to VA-ECMO alone (45). Even though, we do not have prospective or even randomized data supporting the theories on LV venting effects of an additional IABP or transvalvular microaxial flow pump, hemodynamic and experimental studies have proven the underlying theories of LV afterload reduction and therefore the LV decompression (46, 47). At this point it is hard to tell which venting device may be more beneficial in patients with CS. The effectiveness of a transvalvular microaxial flow pump is independent from heart rhythm, rate, and underlying cardiac index. These attributes might contribute to more efficient and sustainable LV venting. On the other hand, an advantage of IABP compared to a transvalvular microaxial flow pump might be a lower bleeding and vascular complication profile.

### Limitations

The most relevant limitation of this study is the lack of any randomized comparison between VA-ECMO and VA-ECMO + IABP. Furthermore, all the included data was collected retrospectively. Most studies do not focus on the comparison between VA-ECMO and VA-ECMO + IABP, therefore the reason why an additional IABP is used is not clear in most studies and selection bias for the use of an IABP might be present in most studies. In addition to the nature of a review, the original studies were thus designed heterogeneously, with potential differences in baseline data and in risk profiles. A further limitation of this study is the small number of patients included in the studies. We also included studies which were published over a considerable period of time, dating back to the last decade. In the meantime, both devices and sheaths used have undergone constant iterative changes, which might have influenced the results.

## Conclusion

In this meta-analysis, combination therapy of VA-ECMO + IABP was superior to VA-ECMO only in patients with CS due to AMI. Randomized and structured data is needed to evaluate the value of VA-ECMO + IABP in patients with CS due to AMI.

## Data availability statement

The original contributions presented in this study are included in the article/supplementary material, further inquiries can be directed to the corresponding author.

## Ethics statement

Ethical review and approval was not required for the study on human participants in accordance with the local legislation and institutional requirements. Written informed consent for participation was not required for this study in accordance with the national legislation and the institutional requirements.

## Author contributions

TT and MM: conceptualization, data curation, formal analysis, investigation, methodology, project administration, validation, visualization, writing—original draft, and writing—review and editing. SM-M, SH, SBr, SN, CA, and SBa: investigation, project administration, resources, supervision, validation, and writing—review and editing. CA and SL: data curation, investigation, project administration, resources, supervision, validation, visualization, writing—original draft, and writing—review and editing. All authors contributed to the article and approved the submitted version.
